# RNA viromes of the oriental hybrid lily cultivar “Sorbonne”

**DOI:** 10.1186/s12864-018-5138-3

**Published:** 2018-10-13

**Authors:** Yeonhwa Jo, Won Kyong Cho

**Affiliations:** Research Institute of Agriculture and Life Sciences, College of Agriculture and Life Sciences, Seoul National University, Seoul, 08826 Republic of Korea

**Keywords:** Genome, Lily, “Sorbonne”, Transcriptome, Virus, Virome

## Abstract

**Background:**

The lily is a perennial flowering plant belonging to the genus *Lilium* in the family *Liliaceae*. Most cultivated lily plants are propagated by bulbs. Therefore, numerous lily bulbs are frequently infected by diverse viruses causing viral diseases. To date, no study has examined the viromes of plants of one type with identical genetic backgrounds collected from different geographical regions.

**Results:**

Here, we examined different viromes of the lily cultivar “Sorbonne” using 172 gigabytes of transcriptome data composed of 23 libraries from four different projects for the cultivar “Sorbonne.” We identified 396 virus-associated contigs from all but one library. We identified six different viruses, including *Plantago asiatica mosaic virus* (PlAMV), *Cucumber mosaic virus* (CMV), *Lily symptomless virus* (LSV), *Tulip virus X* (TVX), *Lily mottle virus* (LMoV), and *Tobacco rattle virus* (TRV). Of them, PlAMV was the most common virus infecting the lily. Scale and flower samples possessed a high number of virus-associated reads. We assembled 32 nearly complete genomes for the six identified viruses possessing the polyadenylate tails. Genomes of all six viruses were highly conserved in the lily cultivar “Sorbonne” based on mutation analysis. We identified defective RNAs from LSV, TVX, and PlAMV localized in the triple gene block region. Phylogenetic analyses showed that virus genomes are highly correlated with geographical regions and host plants.

**Conclusions:**

We conducted comprehensive virome analyses of a single lily cultivar, “Sorbonne,” using transcriptome data. Our results shed light on an array of lily virome-associated topics, including virus identification, the dominant virus, virus accumulation in different plant tissues, virus genome assembly, virus mutation, identification of defective RNAs, and phylogenetic relationships of identified viruses. Taken together, we provide very useful methods and valuable results that can be applied in other virome-associated studies.

**Electronic supplementary material:**

The online version of this article (10.1186/s12864-018-5138-3) contains supplementary material, which is available to authorized users.

## Background

Lilies are tall perennial herbaceous flowering plants, which are members of the genus *Lilium* in the family *Liliaceae* [1]. The flowers of lilies are large, colorful, and very often fragrant. Lilies are cultivated as cut flowers or grown in the garden. Numerous lily cultivars and interspecific hybrids have been developed [2]. To date, there are about 100 known lily species, which can be grouped into seven types [3].

In order to keep the genetic characteristics of lily flowers, lilies are usually clonally propagated using bulbs and scaling, which involves detaching scales from the bulb and planting individual scales to make new bulbs [4]. Due to the clonal propagation of lilies, most lily cultivars are frequently infected by diverse pathogenic microorganisms [5]. Of them, viruses infecting lily species cause serious damage to the quality and quantity of lily production. Since the first report of the three major viruses infecting the lily in 1944 (i.e., *Lily symptomless virus* (LSV) in the genus *Carlavirus*, *Lily mottle virus* (LMoV) in the genus *Potyvirus*, and *Cucumber mosaic virus* (CMV) in the genus *Cucumovirus* [6]), more than 20 viruses infecting the lily have been reported [7].

It is known that LMoV causes vein clearing, leaf mottle, mosaic, and chlorotic symptoms in infected lily plants, whereas LSV-infected plants are generally symptomless when they are grown under normal conditions [8]. In many cases, two or three different viruses infect lilies, resulting in severe economic losses [9]. Viruses infecting lilies are transmitted by mechanical transfer and aphids. For example, many viruses infecting lilies, such as LSV, LMoV, CMV, and *Tulip breaking virus* (TBV) in the genus *Potyvirus*, can be rapidly spread by aphids [8, 10, 11].

In order to diagnose viruses infecting lilies, many studies have developed diverse techniques. For example, a multiplex reverse-transcription (RT) polymerase chain reaction (PCR) [12], a multiplex Luminex bead array [13], a triplex IC-RT-PCR [9], polyclonal antisera [14], RT loop-mediated isothermal amplification [15, 16], and an immunochromatographic strip [17, 18] have been developed. Most studies have focused on the identification of three major viruses: LSV, LMoV, and CMV.

Due to the rapid advances in next-generation sequencing (NGS), co-infected known and novel viruses can be easily identified with full genomes [19–21]. For example, NGS of a small RNA library revealed co-infection of CMV, LSV, and a novel virus referred to as *Lily yellow mosaic virus* (LYMV) in the genus *Potyvirus* [22]. However, the NGS technique is not widely applied in the study of viruses infecting lilies.

Our previous studies have shown that plant transcriptomes are very useful for plant virome studies involving the identification of known and novel viruses, the assembly of virus genomes, mutation analyses, and the comparison of different viromes [23–26]. In this study, we carried out an in silico study of different viromes for the lily cultivar “Sorbonne.” Taking advantage of transcriptome data followed by bioinformatics analyses, we addressed diverse questions associated with lily viromes in this study.

## Results

### Identification of viruses infecting lilies

**Fig. 1:**
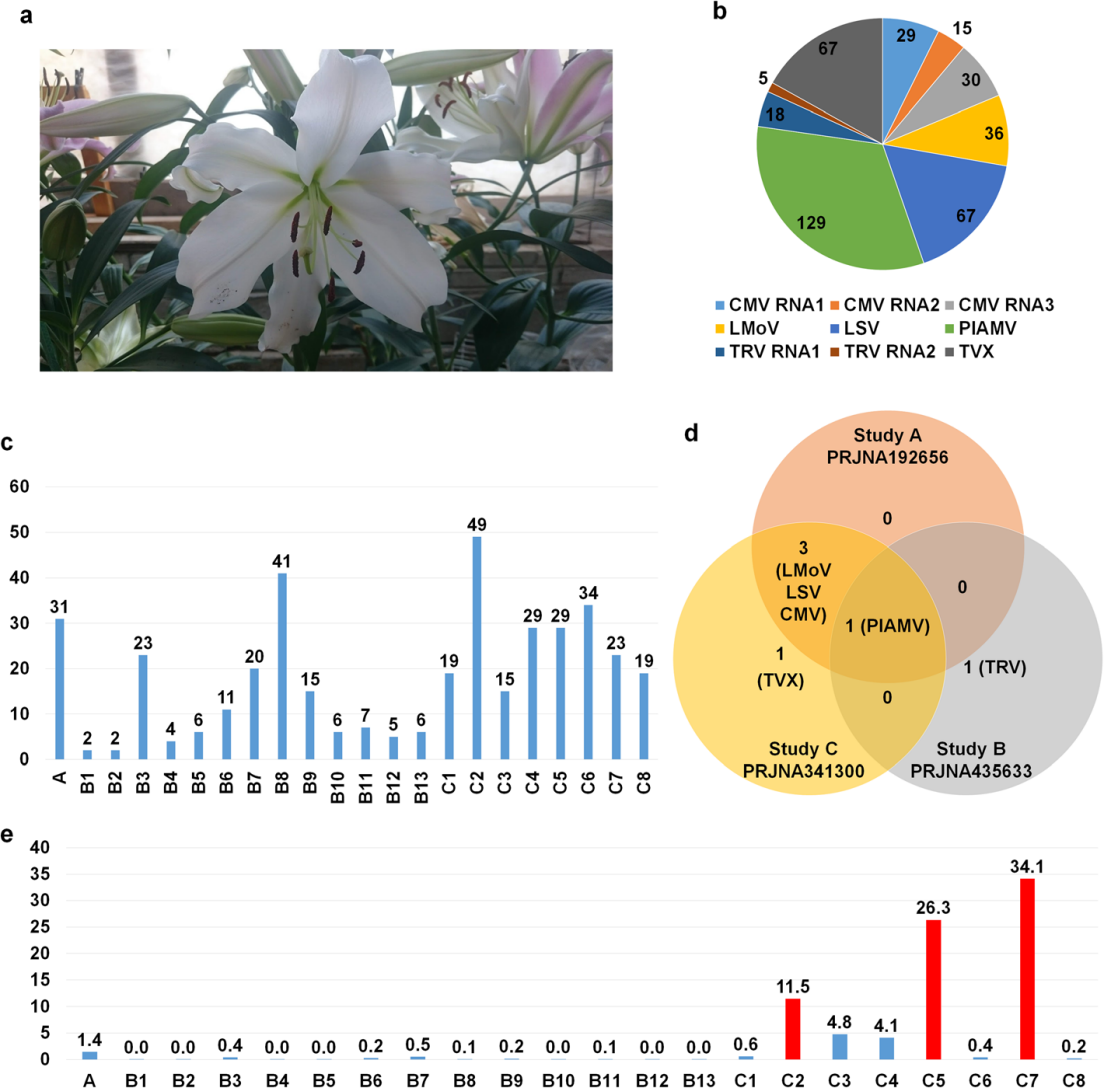
Study of viromes for lily cultivar “Sorbonne”. **a** Flower of lily cultivar “Sorbonne” grown in greenhouse. Image was taken by WKC. **b** Pie chart showing number of virus-associated contigs from all examined transcriptome data. **c** Number of virus-associated contigs in each lily transcriptome. **d** Comparison of identified viruses in three different studies: A, B, and C. **e** Proportion (percentage) of virus-associated reads in each lily transcriptome. Virus-associated reads were obtained by BLASTN search against identified virus reference genome. Red bars indicate lily transcriptomes containing high proportion of virus-associated reads

A total of 23 RNA-Sequencing (RNA-Seq) datasets derived from four different projects were associated with the lily cultivar “Sorbonne” (Fig. 1a). The 23 Sequence Read Archive (SRA) datasets were downloaded and subjected to de novo transcriptome assembly using the Trinity program (Additional file 1: Table S1). Assembled contigs for each library were used for a BLASTN search against the virus reference genome sequences derived from the viral genome database (https://www.ncbi.nlm.nih.gov/genome/viruses/). All but one library (SRR1390677 from PRJNA252055) contained virus-associated contigs. To simplify the names of the three different projects, we created the following names: Study A (PRJNA192656) containing a single library, Study B (PRJNA341300) containing 13 libraries, and Study C (PRJNA435633) containing eight libraries (Table 1). We identified 396 virus-associated contigs representing six viruses from 22 libraries (Fig. 1b). The identified viruses infecting “Sorbonne” were *Plantago asiatica mosaic virus* (PlAMV) and *Tulip virus X* (TVX) in the genus *Potexvirus*, CMV in the genus *Cucumovirus*, LSV in the genus *Carlavirus*, LMoV in the genus *Potyvirus*, and *Tobacco rattle virus* (TRV) in the genus *Tobravirus*. Based on the number of virus-associated contigs, PlAMV (129 contigs) was the dominant virus followed by CMV (74 contigs), LSV (67 contigs), TVX (67 contigs), LMoV (36 contigs), and TRV (23 contigs) (Fig. 1b). The number of virus-associated contigs in each library ranged from two contigs (B1 and B2) to 49 contigs (C2) (Fig. 1c). There were 14 libraries containing more than 10 virus-associated contigs. We next compared the identified viruses among the three different projects (Fig. 1d). Study A contained four viruses: LMoV, LSV, CMV, and PlAMV. Study B contained LMoV, LSV, CMV, PlAMV, and TVX, while Study C contained only two viruses, including PlAMV and TRV. In the case of Study B, we identified PlAMV-associated sequence reads but not assembled contigs. PlAMV was commonly identified in all three studies, while LMoV, LSV, and CMV were commonly identified in Study A and Study B. TVX and TRV were specifically identified in Study B and C, respectively.

**Table 1:** Information of RNA-Seq libraries used for the virome study of lily cultivar “Sorbonne”

Index	SRA accession No.	Tissues
A	SRR787322	Pooled with different tissues such as basal roots, scales, leaves, epidermis, tepals, and stigmas
B1	SRR6837843	Upper stem
B2	SRR6837844	Upper stem
B3	SRR6837845	Lower stem
B4	SRR6837846	Apex
B5	SRR6837847	Upper stem
B6	SRR6837848	Lower stem
B7	SRR6837850	Lower stem
B8	SRR6837851	Root
B9	SRR6837852	Bulb
B10	SRR6837853	Lower stem
B11	SRR6837854	Root
B12	SRR6837855	Leaf
B13	SRR6837856	Upper stem
C1	SRR4125791	Leaf
C2	SRR4128962	Scale
C3	SRR4128968	Scale
C4	SRR4131341	Scale
C5	SRR4131406	Flower
C6	SRR4142853	Leaf
C7	SRR4143329	Flower
C8	SRR4143339	Flower

Name of each library with respective SRA accession number and lily tissues used for library preparation were indicated

### Proportion of virus-associated reads in the transcriptome

Viral abundance can vary depending on sample conditions and library preparation methods. All libraries in the three studies were prepared by mRNA isolation for cDNA library preparation. However, the sample conditions differed. For example, Study A used a mixture of six different tissues, including basal roots, scales, leaves, epidermal tissues, tepals, and stigmas (Table 1). Study B only used aerial bulbils, while Study C used three different tissues, including flowers, leaves, and bulb scales (Table 1). We analyzed the proportion of viral RNA in each library based on read numbers by a BLASTN search (Additional file 1: Table S2). The proportion of viral reads in Study A possessing a single library was 1.4%, whereas the proportion of viral reads in Study B containing 13 libraries ranged from 0 to 0.5% (Fig. 1e). Interestingly, the proportion of viral reads in Study C containing nine libraries was very high, ranging from 0.2 to 34.1%. In particular, samples for C2 (Scale 65) (11.5%), C5 (Flower 62) (26.3%), and C7 (Flower 64) (34.1%) were relatively high. Although there were three biological replicates for each condition, there were strong deviations among the three replicates for viral proportion.

### Viral populations in samples co-infected by different viruses

**Fig. 2:**
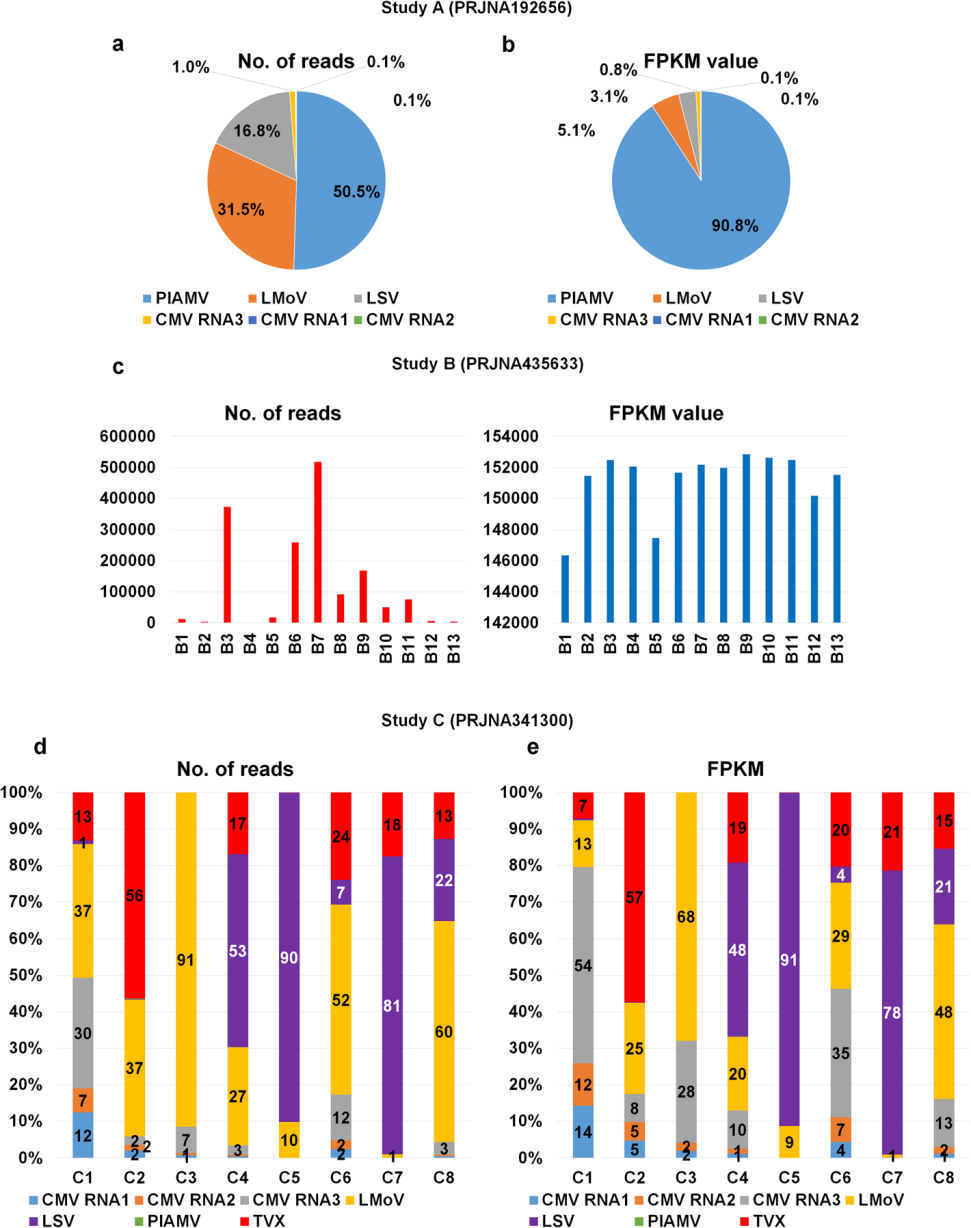
Proportion of identified viruses based on number of virus-associated reads and FPKM values. Pie charts showing proportion of identified viruses based on virus-associated reads (**a**) and FPKM values (**b**) in Study A. All virus-associated reads were identified by BLASTN search against identified virus reference genome. **c** Bar graphs showing change in PlAMV-associated reads (red bar) and FPKM values (blue bar) in different lily transcriptomes for Study B. Bar charts showing proportion of identified viruses based on virus-associated reads (**d**) and FPKM values (**e**) in Study C

As we have shown, most libraries were co-infected by different viruses, except the 12 libraries containing only PlAMV in Study B. To reveal the viral populations in each library, we focused on two different numbers: number of virus-associated reads and number of Fragments Per Kilobase of transcript per Million (FPKM) mapped reads (Additional file 1: Table S2). Virus-associated reads are the total reads associated with the given virus, whereas FPKM values are normalized values using sequencing depth and genome size. In Study A, PlAMV (50.5%) was the dominant virus followed by LMoV (31.5%) and LSV (16.8%) based on the number of reads (Fig. 2a); however, the proportion of PlAMV (90.8%) was dramatically increased based on the FPKM value (Fig. 2b). We examined the proportion of PlAMV in the 13 libraries of Study B (Fig. 2c). FPKM values (blue bar) showed some deviation among libraries; however, the differences were not very significant. On the other hand, the number of reads associated with PlAMV (red bar) in each library changed significantly. In Study C using three different tissues, the virus population in each library changed dramatically. For instance, LMoV was the dominant virus in four libraries, whereas LSV was the dominant virus in three libraries based on the number of reads (Fig. 2d). In contrast, CMV composed of three RNAs was the dominant virus in two libraries (i.e., C1 and C6). In addition, there were strong deviations among the three replicates. For example, three libraries (C2, C3, and C4) were derived from scale samples; however, the viral populations of the three libraries were very different from each other. In RNA-Seq, the number of virus-associated reads is strongly correlated with the virus genome size. Therefore, it is important to use FPKM values that consider the size of the individual virus genome. The proportions of identified viruses in each library based on FPKM values differed from those based on the number of reads. For example, the proportion of CMV RNA3 with a relatively small genome size was dramatically increased in most libraries, such as C1, C3, and C6 (Fig. 2e). In contrast, the proportion of LMoV with a relatively large genome size was strongly decreased in most libraries. For instance, the proportion of LMoV was 91% based on the number of reads; however, the proportion of LMoV was reduced to 68% based on FPKM values in C3.

### Mapping of raw sequence reads and assembly of virus genomes

**Fig. 3:**
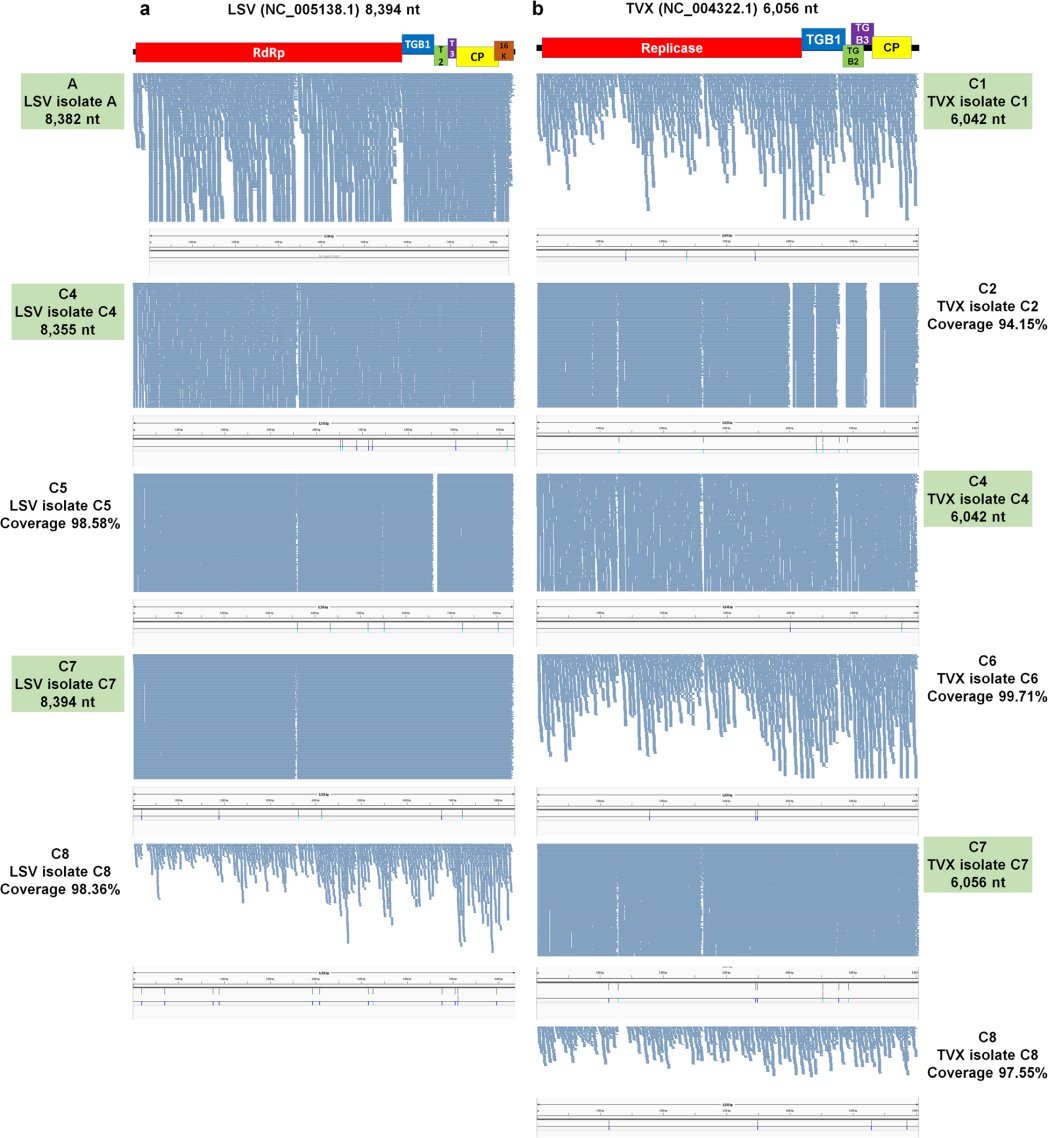
Genome assembly, mapping of reads on virus genome, and mutation analyses for LSV and TVX. Genome organization of LSV (**a**) and TVX (**b**) based on corresponding reference genome. Green boxes indicate information of assembled virus genome, such as SRA number, name of isolate, and genome size. The positions of identified SNPs in each virus genome were visualized by the Tablet program

**Fig. 4:**
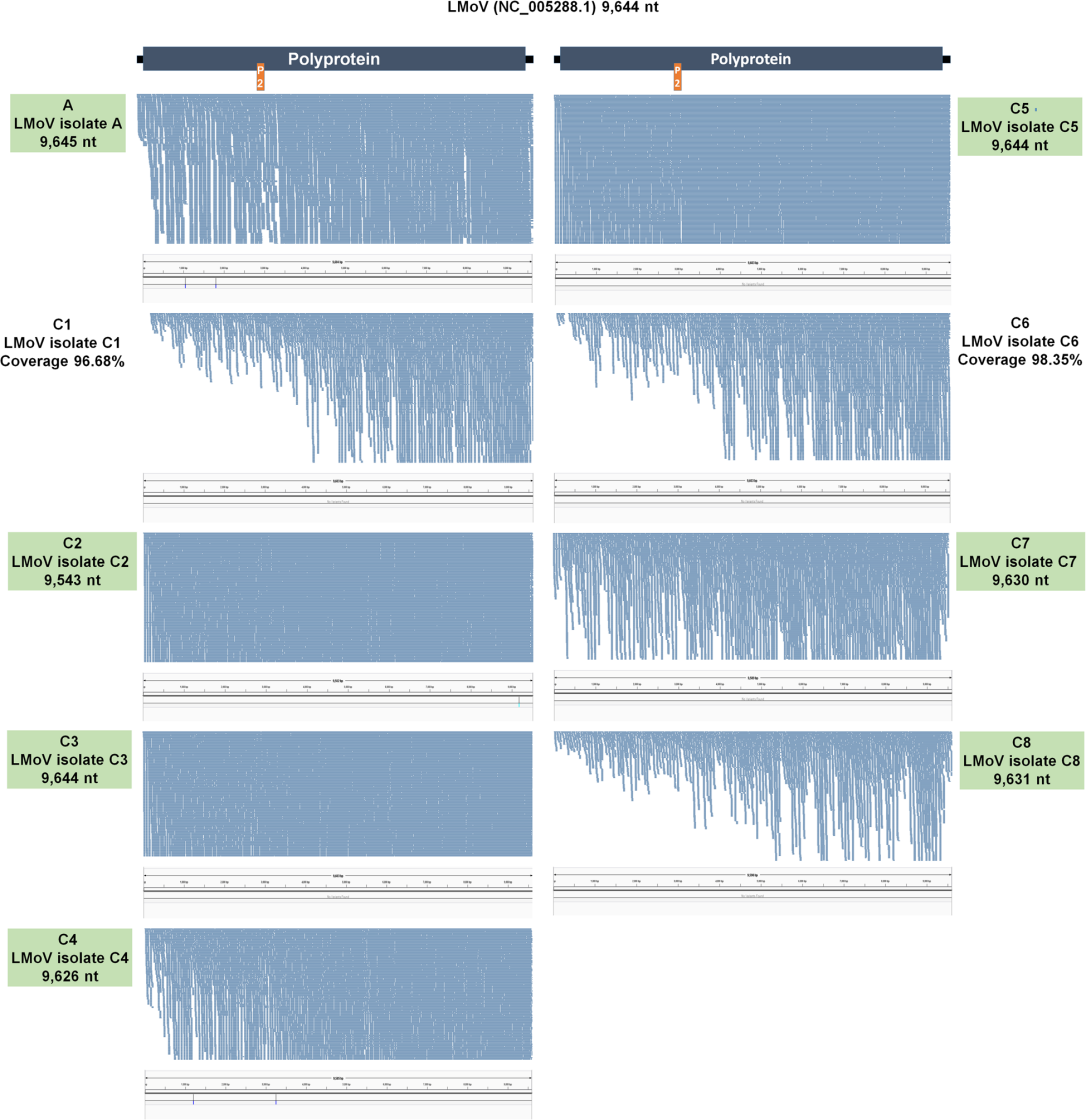
Genome assembly, mapping of reads on virus genome, and mutation analyses for LMoV. Genome organization of LMoV based on corresponding reference genome. Green boxes indicate information of assembled virus genome, such as SRA number, name of isolate, and genome size. The positions of identified SNPs in each virus genome were visualized by the Tablet program

**Fig. 5:**
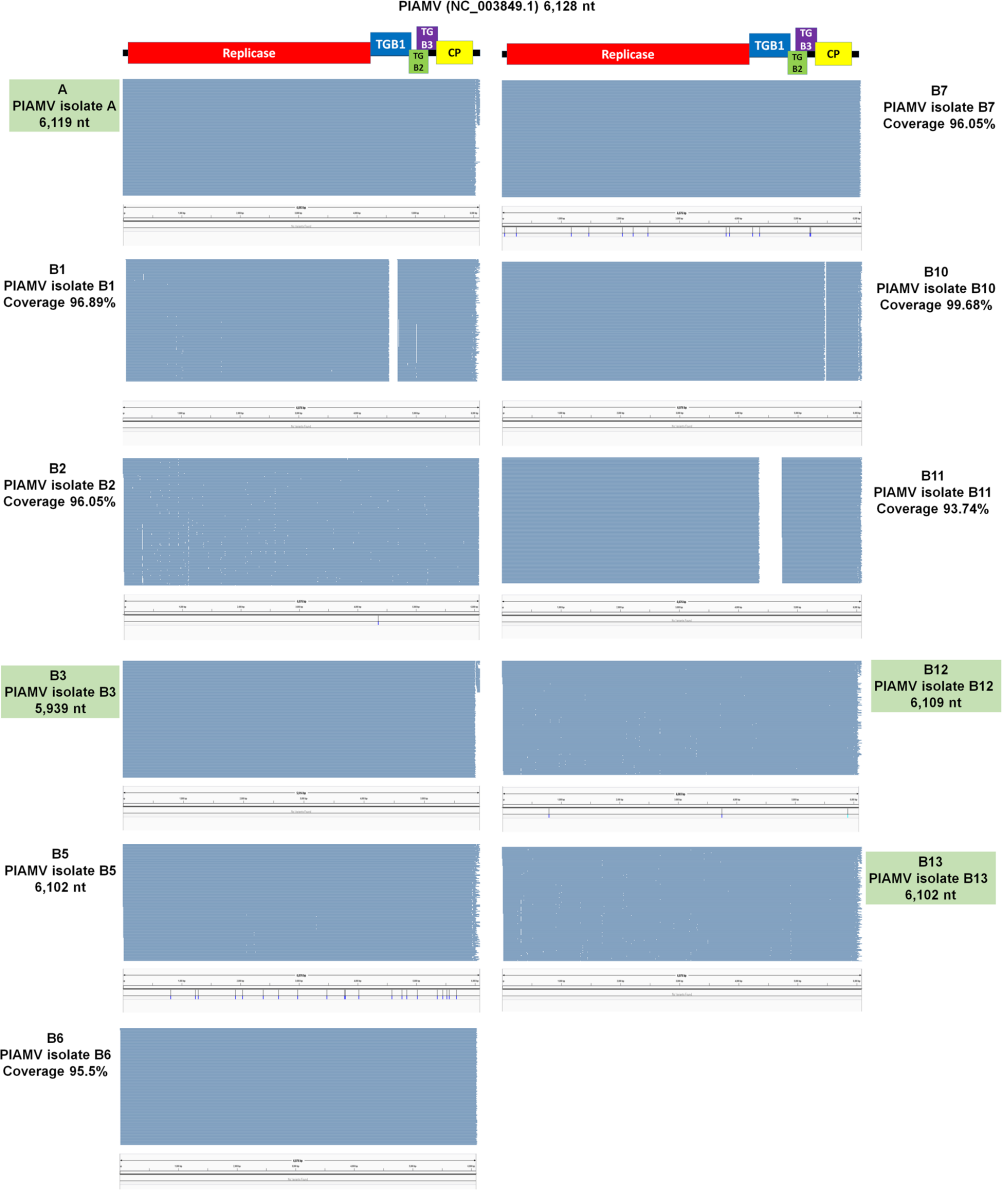
Genome assembly, mapping of reads on virus genome, and mutation analyses for PlAMV. Genome organization of PlAMV based on corresponding reference genome. Green boxes indicate information of assembled virus genome, such as SRA number, name of isolate, and genome size. The positions of identified SNPs in each virus genome were visualized by the Tablet program

**Fig. 6:**
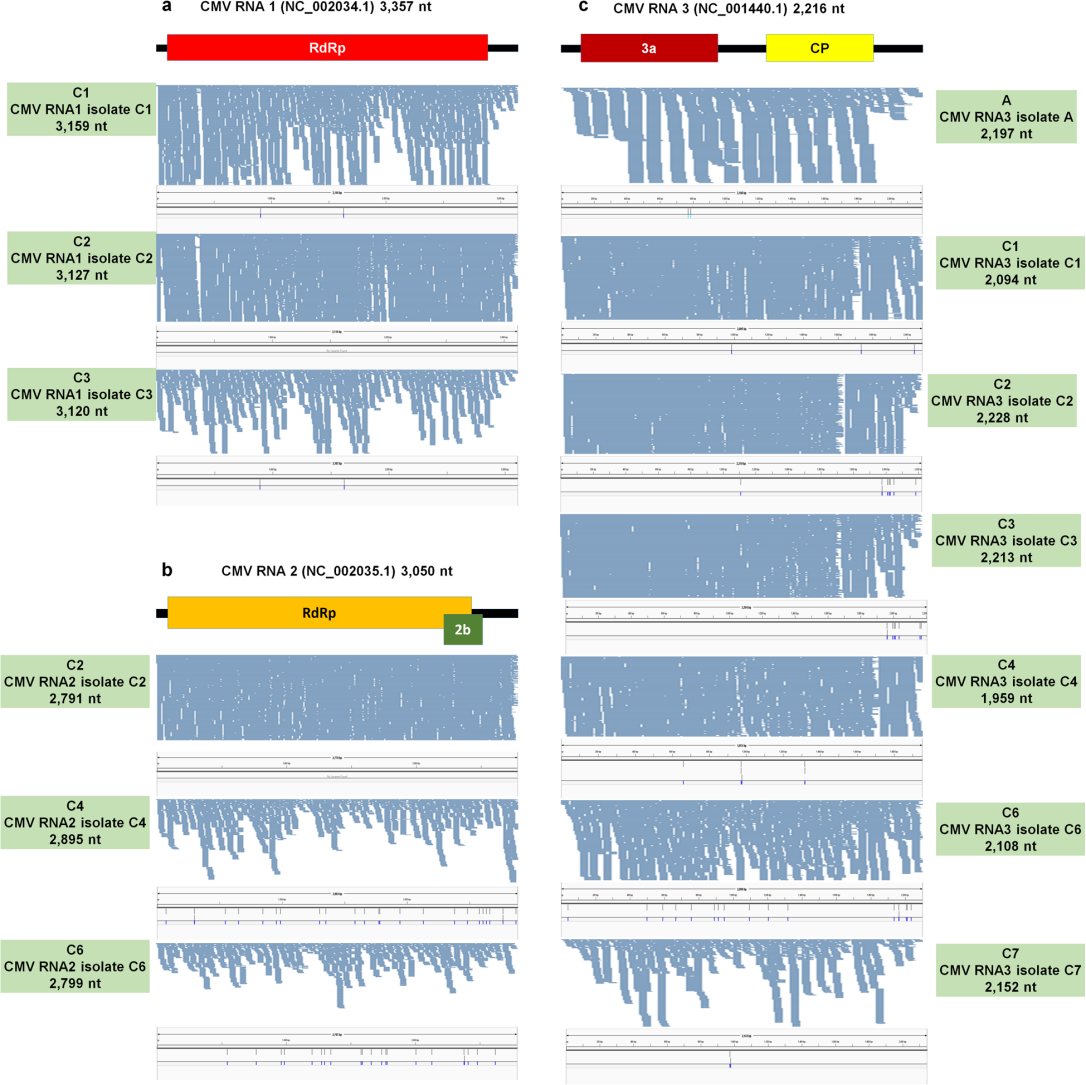
Genome assembly, mapping of reads on virus genome, and mutation analyses for CMV. Genome organization of three RNA fragments, RNA 1 (**a**), RNA 2 (**b**), and RNA 3 (**c**), of CMV genome. Green boxes indicate information of assembled virus genome, such as SRA number, name of isolate, and genome size. The positions of identified SNPs in each virus genome were visualized by the Tablet program

**Fig. 7:**
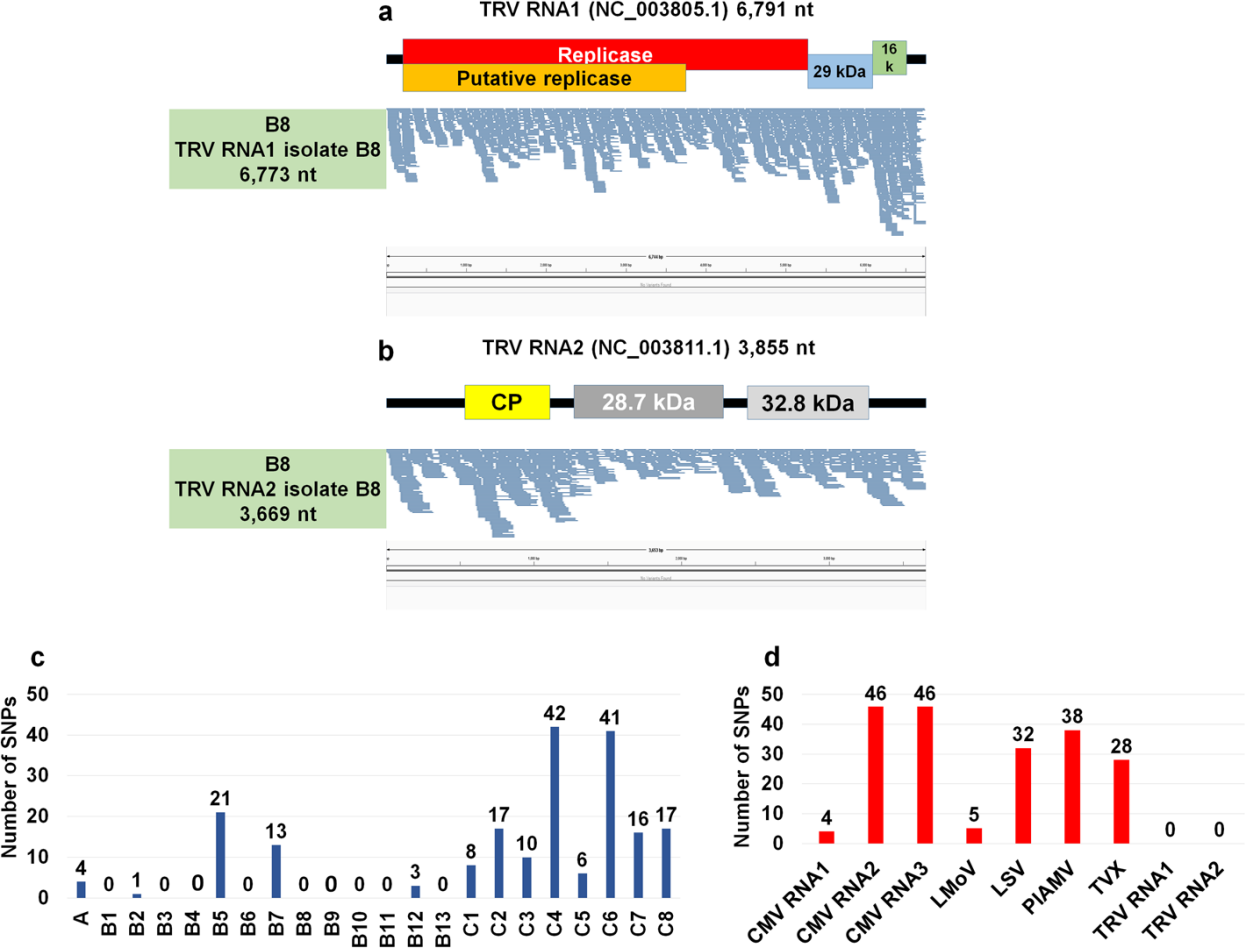
Genome assembly, mapping of reads on virus genome, and mutation analyses for TRV and number of identified SNPs. Genome organization of two RNA fragments, RNA1 (**a**) and RNA2 (**b**), of TRV genome. Green boxes indicate information of assembled virus genome, such as SRA number, name of isolate, and genome size. The positions of identified SNPs in each virus genome were visualized by the Tablet program. Number of identified SNPs associated with identified viruses in each transcriptome (**c**) and total number of identified SNPs for each virus (**d**)

The sizes of virus-associated contigs ranged from 201 bp to 9661 bp (Table 2). Of the identified viruses, many contigs for LMoV were larger than 9000 bp, meaning they could nearly cover the complete genome of LMoV. In order to assemble the genomes of the identified viruses, we mapped raw sequence reads on each virus reference genome (Figs. 3, 4, 5, 6, 7). By mapping, sequence coverage of several identified virus isolates (e.g., five isolates for LSV, five isolates for TVX, nine isolates for LMoV, 11 isolates for PlAMV, three isolates for CMV RNA1, three isolates for CMV RNA2, seven isolates for CMV RNA3, one isolate for TRV RNA1, and one isolate for TRV RNA2) was more than 90%. In many cases, most regions of the target virus genome were fully mapped by virus-associated reads; however, some specific regions in some viruses were not mapped. Moreover, the number of mapped reads on LMoV was increased from the 5′ region to the 3′ region (Fig. 4). In the case of LSV isolate C5 with 98.58% coverage, a partial triple gene block (TGB) region was not mapped (Fig. 3a). Again, in TVX isolate C2 with 94.15% coverage, many TGB regions were not mapped (Fig. 3b). Moreover, three PlAMV isolates (i.e., B1, B10, and B11) contained unmapped regions (Fig. 5). Based on mapping and assembled virus-associated contigs, we obtained 32 nearly complete genomes: three genomes for CMV RNA1, three genomes for CMV RNA2, seven genomes for CMV RNA3, seven genomes for LMoV, three genomes for LSV, four genomes for PlAMV, one genome for TRV RNA1, one genome for TRV RNA1, and three genomes for TVX (Table 3). Genomes of some virus isolates lacking specific regions were not included.

**Table 2:** Summary of identified virus-associated contigs

Study	Project No.	SRA No.	Virus	No. of contigs	Minimum contig length (bp)	Maximum contig length (bp)
Study A	PRJNA192656	SRR787322	PlAMV	4	6140	6149
Study A	PRJNA192656	SRR787322	LMoV	2	9656	9661
Study A	PRJNA192656	SRR787322	LSV	4	3611	4775
Study A	PRJNA192656	SRR787322	CMV RNA3	3	289	2196
Study A	PRJNA192656	SRR787322	CMV RNA1	12	262	731
Study A	PRJNA192656	SRR787322	CMV RNA2	6	315	1013
Study B	PRJNA435633	SRR6837843	PlAMV	2	1562	4501
Study B	PRJNA435633	SRR6837844	PlAMV	2	788	5112
Study B	PRJNA435633	SRR6837845	PlAMV	23	204	5227
Study B	PRJNA435633	SRR6837846	PlAMV	4	382	1412
Study B	PRJNA435633	SRR6837847	PlAMV	6	232	3790
Study B	PRJNA435633	SRR6837848	PlAMV	11	275	4485
Study B	PRJNA435633	SRR6837850	PlAMV	20	201	5434
Study B	PRJNA435633	SRR6837851	PlAMV	18	277	2715
Study B	PRJNA435633	SRR6837851	TRV RNA2	5	1224	3669
Study B	PRJNA435633	SRR6837851	TRV RNA1	18	218	6773
Study B	PRJNA435633	SRR6837852	PlAMV	15	214	4188
Study B	PRJNA435633	SRR6837853	PlAMV	6	232	3861
Study B	PRJNA435633	SRR6837854	PlAMV	7	237	3729
Study B	PRJNA435633	SRR6837855	PlAMV	5	818	6145
Study B	PRJNA435633	SRR6837856	PlAMV	6	779	4446
Study C	PRJNA341300	SRR4125791	CMV RNA3	2	917	1237
Study C	PRJNA341300	SRR4125791	CMV RNA1	2	411	2774
Study C	PRJNA341300	SRR4125791	CMV RNA2	1		2688
Study C	PRJNA341300	SRR4125791	TVX	6	312	2162
Study C	PRJNA341300	SRR4125791	LSV	6	201	855
Study C	PRJNA341300	SRR4125791	LMoV	2	392	8952
Study C	PRJNA341300	SRR4128962	TVX	22	205	1442
Study C	PRJNA341300	SRR4128962	LMoV	12	332	5408
Study C	PRJNA341300	SRR4128962	CMV RNA3	3	201	1762
Study C	PRJNA341300	SRR4128962	CMV RNA1	2	378	2768
Study C	PRJNA341300	SRR4128962	CMV RNA2	1		2791
Study C	PRJNA341300	SRR4128962	LSV	9	231	2183
Study C	PRJNA341300	SRR4128962	PlAMV	0		
Study C	PRJNA341300	SRR4128968	LMoV	7	282	5360
Study C	PRJNA341300	SRR4128968	CMV RNA3	4	514	985
Study C	PRJNA341300	SRR4128968	CMV RNA1	3	297	2751
Study C	PRJNA341300	SRR4128968	CMV RNA2	1		2686
Study C	PRJNA341300	SRR4131341	LSV	8	205	3626
Study C	PRJNA341300	SRR4131341	LMoV	2	4302	5349
Study C	PRJNA341300	SRR4131341	TVX	9	276	1914
Study C	PRJNA341300	SRR4131341	CMV RNA3	5	246	873
Study C	PRJNA341300	SRR4131341	CMV RNA1	3	376	1708
Study C	PRJNA341300	SRR4131341	CMV RNA2	2	913	2004
Study C	PRJNA341300	SRR4131406	LSV	10	202	2593
Study C	PRJNA341300	SRR4131406	LMoV	4	1993	7253
Study C	PRJNA341300	SRR4131406	TVX	10	239	962
Study C	PRJNA341300	SRR4131406	CMV RNA3	5	201	352
Study C	PRJNA341300	SRR4142853	LMoV	3	207	8811
Study C	PRJNA341300	SRR4142853	TVX	5	350	2141
Study C	PRJNA341300	SRR4142853	CMV RNA3	2	898	1251
Study C	PRJNA341300	SRR4142853	LSV	15	210	1019
Study C	PRJNA341300	SRR4142853	CMV RNA2	3	829	1054
Study C	PRJNA341300	SRR4142853	CMV RNA1	6	205	795
Study C	PRJNA341300	SRR4143329	LSV	9	211	2604
Study C	PRJNA341300	SRR4143329	TVX	9	312	2018
Study C	PRJNA341300	SRR4143329	LMoV	1		9631
Study C	PRJNA341300	SRR4143329	CMV RNA3	3	432	924
Study C	PRJNA341300	SRR4143329	CMV RNA1	1		217
Study C	PRJNA341300	SRR4143329	CMV RNA2	0		
Study C	PRJNA341300	SRR4143339	LMoV	3	271	9003
Study C	PRJNA341300	SRR4143339	LSV	6	201	2547
Study C	PRJNA341300	SRR4143339	TVX	6	304	2137
Study C	PRJNA341300	SRR4143339	CMV RNA3	3	254	1001
Study C	PRJNA341300	SRR4143339	CMV RNA2	1		332
Study C	PRJNA341300	SRR4143339	CMV RNA1	0		

Name of identified virus, number of virus-associated contigs, and lengths of minimum and maximum contigs, respectively, are indicated

**Table 3:** List of assembled virus genomes

Index	Virus genome	Study	Size (nt)	Accession No.
1	CMV-RNA1	C1	3159	MH360227
2	CMV-RNA1	C2	3127	MH360228
3	CMV-RNA1	C3	3120	MH360229
4	CMV-RNA2	C2	2791	MH360230
5	CMV-RNA2	C4	2895	MH360231
6	CMV-RNA2	C6	2799	MH360232
7	CMV-RNA3	A	2197	MH360233
8	CMV-RNA3	C1	2094	MH360234
9	CMV-RNA3	C3	2213	MH360235
10	CMV-RNA3	C4	1959	MH360236
11	CMV-RNA3	C6	2108	MH360237
12	CMV-RNA3	C7	2152	MH360238
13	LMoV	A	9645	MH360239
14	LMoV	C2	9543	MH360240
15	LMoV	C3	9644	MH360241
16	LMoV	C4	9626	MH360242
17	LMoV	C5	9644	MH360243
18	LMoV	C7	9630	MH360244
19	LMoV	C8	9631	MH360245
20	LSV	A	8382	MH360246
21	LSV	C4	8355	MH360247
22	LSV	C7	8394	MH360248
23	PlAMV	A	6119	MH360249
24	PlAMV	B5	6102	MH360250
25	PlAMV	B12	6109	MH360251
26	PlAMV	B13	6102	MH360252
27	TRV-RNA1	B8	6773	MH360253
28	TRV-RNA2	B8	3669	MH360254
29	TVX	C1	6042	MH360255
30	TVX	C4	6042	MH360256
31	TVX	C7	6056	MH360257
32	CMV-RNA3	C2	2208	MH360258

De novo-assembled virus genome in each library, genome size, and respective accession numbers are provided

### Mutation rates of identified viruses

Most RNA viruses exhibit strong genetic variation within the infected host. In order to examine mutation positions and rates for identified viruses, we analyzed single nucleotide polymorphisms (SNPs) using assembled virus genome sequences as references. For example, the assembled viral genome of LSV isolate A was used for SNP calling of LSV genome in the library A while the assembled genome of LSV isolate C4 was used for SNP calling of LSV genome in the library C4. In the case of LSV, we identified no SNP in isolate A, seven SNPs in isolate C4, six SNPs in isolate C5, six SNPs in isolate C7, and 12 SNPs in isolate C8 (Fig. 3a). In TVX, we identified three SNPs in C1, six SNPs in C2, two SNPs in C4, three SNPs in C6, seven SNPs in C7, and four SNPs in C8 (Fig. 3b). In the case of LMoV, six isolates (C1, C3, C5, C6, C7, and C8) did not contain any SNP, while isolate A (two SNPs), isolate C2 (one SNP), and isolate C4 (two SNPs) possessed a small number of SNPs (Fig. 4). In the case of PlAMV, seven out of 11 isolates did not contain any SNP, while two, isolate B5 (21 SNPs) and isolate B7 (13 SNPs), contained several SNPs (Fig. 5). We also identified several SNPs in CMV RNAs (Fig. 6). For example, three were two SNPs for CMV RNA1 of isolate C1 and C3 (Fig. 6a). In the case of CMV RNA2, we identified 23 SNPs in isolate C4 and 20 SNPs in C5 (Fig. 6b). In CMV RNA3, we identified a relatively large number of SNPs as compared to the other two RNAs (Fig. 6c). In particular, isolates C2 and C3 contained many SNPs in the 3′ region. Of the seven CMV RNA genomes examined, all isolates contained at least one SNP (Fig. 6). There were no SNPs in TRV RNA1 (Fig. 7a) or RNA2 (Fig. 7b). Of the 22 libraries examined, nine libraries did not contain any SNPs, while some libraries, such as B5 (21 SNPs), C4 (42 SNPs), and C6 (41 SNPs), contained many SNPs (Fig. 7c). Based on each virus genome, CMV RNA2 and CMV RNA3 contained the largest number of SNPs (46 SNPs), while CMV RNA1 (four SNPs), LMoV (five SNPs), and two TRV RNA genomes (0 SNPs) did not contain any SNPs (Fig. 7d).

### Virus genome assembly and phylogenetic analyses

We obtained 32 nearly complete genomes for all six identified viruses using the obtained virus-associated contigs and mapping of reads on the corresponding reference genome sequences (Table 3 and Additional file 1: Table S3). All obtained virus genome sequences were deposited in GenBank with respective accession numbers. In the case of CMV composed of three different RNAs, three CMV RNA1, three RNA2, and seven RNA3 genome sequences were obtained (Table 3). Seven genomes for LMoV, three genomes for LSV, four genomes for PlAMV, and three genomes for TVX were identified. In addition, we obtained a nearly complete genome for TRV containing two RNA fragments.

**Fig. 8:**
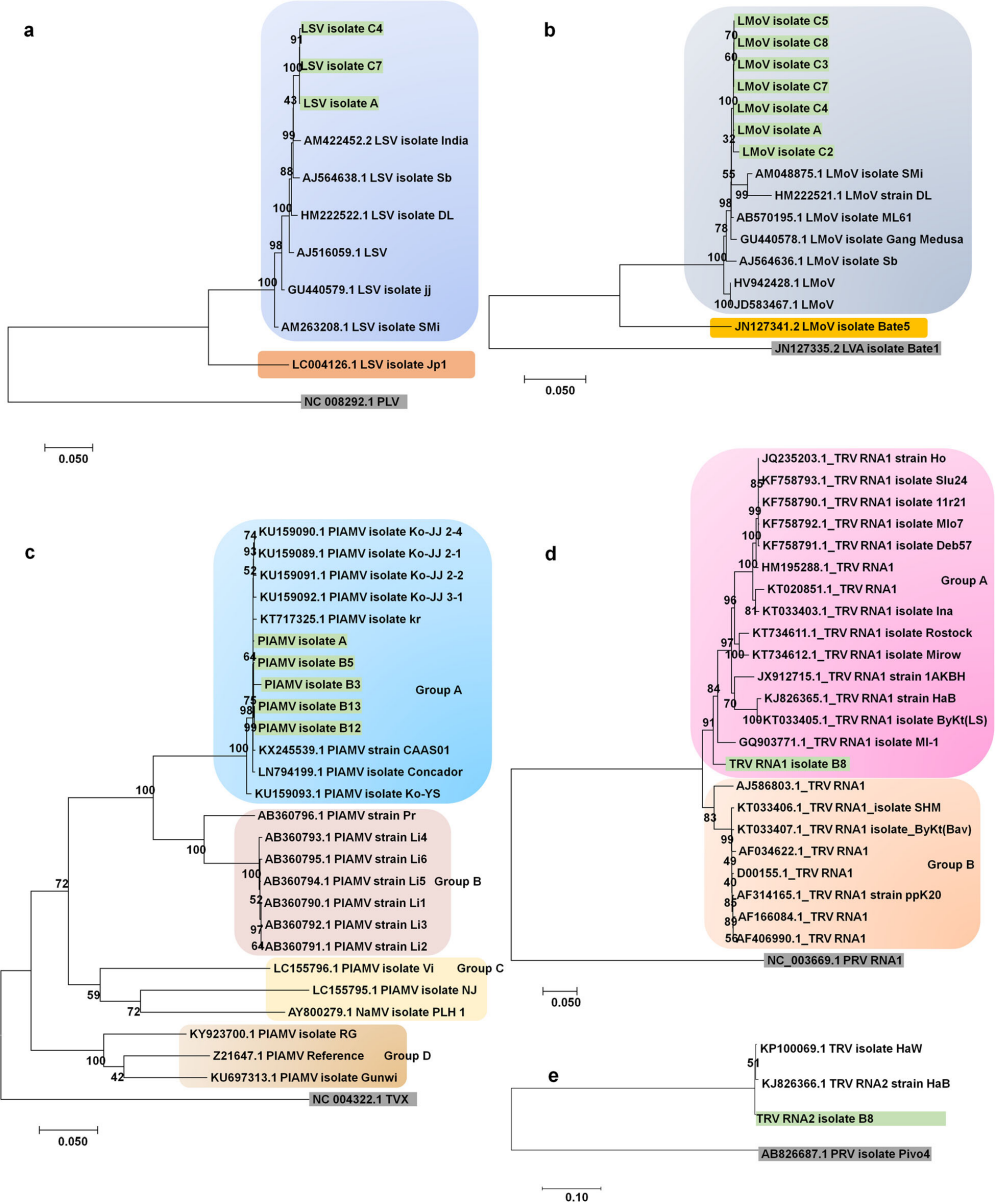
Phylogenetic relationships of LSV, LMoV, PlAMV, and TRV isolates with known isolates. **a** Phylogenetic tree of complete genomes for three LSV isolates, A, C4, and C7. *Potato latent virus* (PLV) was used as an outgroup. **b** Phylogenetic tree of complete genomes for seven LMoV isolates, A, C2, C3, C4, C5, C7, and C8. *Lily virus A* (LVA) was used as an outgroup. **c** Phylogenetic tree of complete genomes for five PlAMV isolates, A, B3, B5, B12, and B13. TVX was used as an outgroup. Phylogenetic tree of complete RNA1 (**d**) and RNA2 (**e**) for TRV isolate B8. *Pepper ringspot virus* (PRV) was used as an outgroup. Available genome sequences for LSV, LMoV, PlAMV, and TRV were also used for phylogenetic construction. Accession numbers and names of virus isolates or strains were provided. The isolates from this study were indicated by the color green

**Fig. 9:**
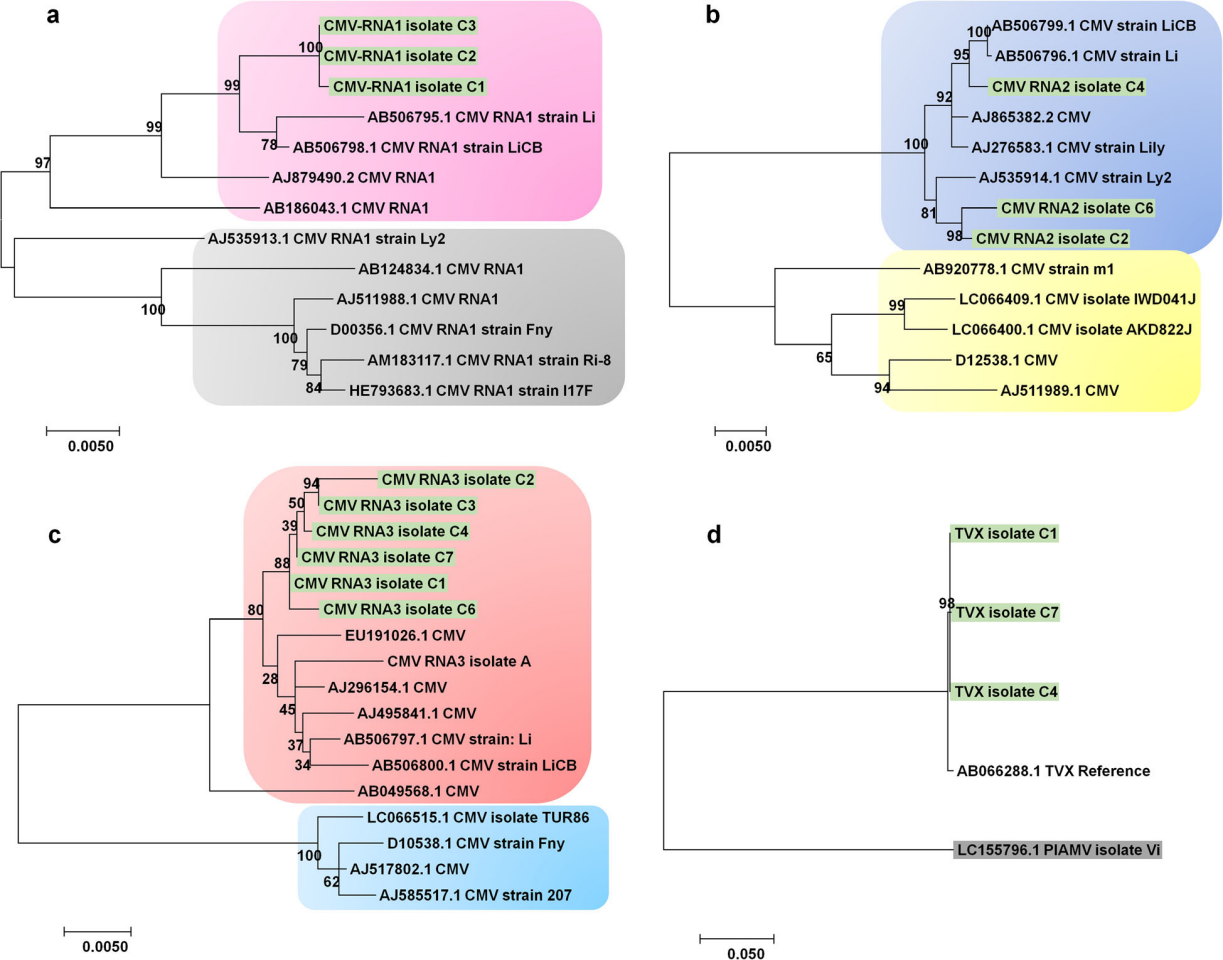
Phylogenetic relationships of CMV and TVX with known isolates. Phylogenetic trees of RNA1 (**a**), RNA2 (**b**), and RNA2 (**c**) for identified CMV isolates. Due to the presence of a large number of CMV genome sequences, only highly matched CMV isolates were used for BLASTN search. **d** Phylogenetic tree of TVX isolates, C1, C4, and C7. PlAMV was used as an outgroup. Available genome sequences for CMV and TVX were also used for phylogenetic construction. Accession numbers and names of virus isolates or strains were provided. The isolates from this study were indicated by the color green

We next generated phylogenetic trees for the identified viruses using assembled genome sequences as well as publicly available virus genome sequences. A phylogenetic tree using 10 LSV genomes, nine LSV genomes including three isolates in this study is closely related except LSV isolate Jp1 (Fig. 8a). All nine LSV genomes were derived from Korea and China, while LSV isolate Jp1 originated from Japan. Similarly, the phylogenetic tree for 15 LMoV isolates showed two different phylogenetic clades (Fig. 8b). The first clade contained seven LMoV isolates from this study and seven LMoV isolates from previous studies. All 14 LMoV isolates have been identified from lilies in China, Korea, and Japan except LMoV isolate Bate5 derived from lilies in Australia [27]. The phylogenetic tree for PlAMV displayed four different groups of PlAMV isolates (Fig. 8c). Group A contained 13 PlAMV isolates, including five isolates in this study mostly derived from China and Korea, except isolate Concador from Hungary. Group B possessed seven isolates, all from Japan. PlAMV isolates in Groups C and D were highly divergent as compared to those in Groups A and B, which were derived from lilies. PlAMV isolates in Group C were derived from *Viola grypoceras* and *Nandina domestica,* while PlAMV isolates in Group D were derived from *Rehmannia glutinosa*, plantain, and *Plantago asiatica*. In the case of TRV possessing two RNA fragments, we generated two different phylogenetic trees (Fig. 8d and e). The phylogenetic tree using TRV RNA1 sequences showed two clades, Group A and Group B. Group A contained 15 TRV isolates along with isolate B8 from this study, whereas Group B included eight TRV isolates. In the case of TRV RNA2, there were only three TRV RNA2 sequences derived from tobacco and isolate B8 from lilies (Fig. 8e). In the case of CMV composed of three RNA fragments, there were numerous genome sequences. Therefore, only the best-matching genome sequences were used for phylogenetic tree construction (Fig. 9a, b, and c). Interestingly, all three and six isolates for CMV RNA1 and RNA3, respectively, were grouped together (Fig. 9a and c). In the case of CMV RNA2, all three isolates in this study were grouped together; other CMV isolates were from lilies. At the time of this study, there was only one TVX genome sequence available (Fig. 9d). The TVX genome sequence from this study showed strong genetic similarity to the TVX reference sequence from a tulip in Japan [28].

## Discussion

A virome is defined as a collection of nucleic acids that constitute viruses in a particular organism or environment [29]. Several previous studies on plant viromes have examined viruses using samples collected from diverse hosts or restricted regions [30, 31]. Moreover, some plant virome studies have examined viruses infecting single cultivars of diverse horticulture plants [23, 25, 26]. However, to date, no study has examined the viromes of plants of one type with identical genetic backgrounds collected from different geographical regions. Thus, we examined the different viromes of the lily cultivar “Sorbonne,” which is clonally propagated by bulbs and widely grown throughout the world.

Our first question was focused on whether the same plant cultivar has identical viromes among different plants grown in diverse regions. Our study clearly answered that question, in that the viromes of the lily cultivar “Sorbonne” were different, although the viruses, such as PlAMV, were commonly identified in three studies. This result demonstrated that some viruses might be transmitted by clonal propagation; however, other viruses could be newly infected under different environmental conditions (e.g., through co-cultivated plants or insect vectors). Thus, plants, viruses, and environments are important factors affecting the viromes of specific plants, as suggested previously [32].

Our next question was focused on how many viruses can infect a single lily plant. Our study showed that up to five different viruses (LMoV, LSV, CMV, TVX, and PlAMV) can infect a single lily plant. To date, LMoV, LSV, and CMV are the most common viruses infecting lilies, while PlAMV resulting in serious damage to lily production is considered one of the rapidly spreading viruses in *Lilium* species [33, 34]. Although cases of TVX and TRV infecting lilies have been reported [35–37], they have not been as well reported as the other four viruses. In addition, we did not find any novel viruses in our study.

NGS or high-throughput sequencing (HTS) is now the most important technique for plant virus diagnostics [38] and virus discovery [39]. In fact, virome studies using NGS not only reveal the presence of viruses but also provide nucleotide sequences associated with the identified viruses. The information of virus-associated sequences is a key factor for virome studies, illustrating virus genome assembly, number of virus-associated sequence reads, and virus mutation, as shown previously [23, 25, 26]. For instance, the choice of plant tissues and developmental stages is important for plant virus diagnostics using molecular methods. Interestingly, our study demonstrated that transcriptomes from scale and flower samples possessed high numbers of virus-associated reads, suggesting that those tissues are much more useful than normal leaves for virus diagnostics, at least in lilies.

Co-infection of diverse viruses in a single plant is now common in many horticulture plants. Furthermore, it is difficult to determine which virus is the main virus causing disease symptoms. Nevertheless, it is now possible to identify a dominant virus with virus-associated reads using transcriptome data. As indicated in our previous study [24, 26], this is done by using normalized values, such as FPKM, to calculate the proportions of individual viruses. With the co-infection of LMoV, LSV, CMV, and PlAMV in Study A, PlAMV was the dominant virus, suggesting a possible main virus causing disease symptoms. However, co-infection of five viruses in different tissues showed variable proportions of diverse viruses. Based on this result, we carefully hypothesized that different tissues and developmental stages affect virus populations.

Using virus-associated contigs and reads, we assembled nearly complete genomes of several viruses. Interestingly, all six viruses possessed the polyadenylate tails [40]. Therefore, we suggested that the possession of poly(A) tails in all six viruses might facilitate the assembly of virus genomes. The assembled genomes were used as reference genomes for virus mutation analysis and used for phylogenetic analyses. As compared to our previous studies [25, 26], genomes of identified viruses were highly conserved. Some viruses showed mutations; however, the mutation rates were very low, and the mutation positions in the genome were mostly random. In addition, a small number of SNPs might be associated with sequencing errors. Thus, we carefully hypothesized that all six viruses were highly conserved, at least in the lily cultivar “Sorbonne.” It is likely that the mutation rates of identified viruses might be different in other lily cultivars or under other environmental conditions.

Mapping of raw sequence reads on the viral genome revealed that three viruses (LSV, TVX, and PlAMV) produced defective RNAs. Interestingly, genomes of all three viruses had three partially overlapping open reading frames (ORFs) referred to as TGB, which is common in the order of *Tymovirales*. Moreover, some isolates produced defective RNAs that were preferentially localized in the TGB region. A previous study demonstrated that the defective RNA produced from *Potato mop-top virus* (PMTV) in the genus *Pomovirus* interferes with virus infection [41]. A recent study also revealed that defective RNA from *Tomato black ring virus* (TBRV) in the genus *Nepovirus* interfered with TBRV replication [42]. Thus, we suggested that the identified defective RNAs in our study could be defective interfering (DI) RNAs, which might be produced by the error-prone viral replicase [43]. However, the possible role of defective RNAs in our study as interfering RNAs should be further experimentally characterized.

With the help of the NGS technique, numerous virus genomes are now being assembled [44]. Similarly, we obtained 32 nearly complete virus genomes, which were further used for phylogenetic analyses. Our results showed that virus genomes are highly correlated with geographical regions. For instance, LSV isolates from Korea and China were closely related with one another but not with an isolate from Japan. Similarly, PlAMV isolates from China and Korea were grouped together, while PlAMV isolates from Japan were clustered together. However, LMoV isolates from China, Korea, and Japan were clustered together. Furthermore, PlAMV isolates from lilies were highly divergent as compared to those from other host plants, suggesting that plant host type is an important factor in distinguishing different virus isolates.

## Conclusions

We conducted comprehensive virome analyses of a single lily cultivar, “Sorbonne,” using transcriptome data. Our results shed light on an array of lily virome-associated topics, including virus identification, the dominant virus, virus accumulation in different plant tissues, virus genome assembly, virus mutation, identification of defective RNAs, and phylogenetic relationships of identified viruses. Taken together, we provide very useful methods and valuable results that can be applied in other virome-associated studies.

## Methods

### Collection of RNA-Seq data for lily cultivar “Sorbonne” from SRA database

We searched RNA-Seq data for the lily cultivar “Sorbonne” in the SRA database of the National Center for Biotechnology Information (NCBI) (https://www.ncbi.nlm.nih.gov/sra) using “Lily” and “Sorbonne” as queries. There were 23 RNA-Seq datasets from four different projects. Detailed information on the RNA-Seq datasets can be found in Additional file 1: Table S1. Moreover, all detailed information on plant materials, library preparation, and RNA-Seq in each project can be found in the previous studies. Each library was newly indexed for simplicity based on the project. PRJNA192656, indexed as A, contained a single SRA dataset that was prepared from a mixture of six different tissues, including basal roots, scales, leaves, epidermal tissues, tepals, and stigmas for total RNA extraction [45]. PRJNA435633, indexed as B1 to B13, contained 13 SRA datasets that were prepared from different tissues. PRJNA341300, indexed as C1 to C8, included eight SRA datasets that were prepared from three different tissues (leaves, flowers, and scales) at different developmental stages [46]. The samples in Study A were derived from the gardens of the plants that were grown in the gardens of the institute of landscape architecture, Zhejiang University, Hangzhou, China. The samples in Study B were derived from the Institute of Botany, Chinese Academy of Sciences in Beijing, China. The samples in Study C were derived from Shanxi Agricultural University, Shanxi, China. We used a workstation with two 20-core CPUs and 256 GB of RAM installed with Ubuntu 16.04.4 LTS for all data analyses. All 23 SRA datasets were downloaded from the SRA database with corresponding accession numbers using the SRA toolkit (https://www.ncbi.nlm.nih.gov/sra/docs/toolkitsoft/). All downloaded SRA datasets were converted to fastq files using fastq-dump implemented in the SRA toolkit.

### De novo transcriptome assembly and identification of virus-associated contigs

In order to identify virus-associated contigs in each RNA-Seq library, bioinformatics analyses were conducted as described previously [26]. In brief, single-end or paired-end sequenced fastq files in each library were subjected to de novo transcriptome assembly using the Trinity program (v2.6.5 Release) with default parameters [47]. All assembled contigs (transcriptomes) in each library were used for MEGABLAST [48] with a cut-off E-value of 1e^− 6^ to search against NCBI’s viral reference database downloaded from https://www.ncbi.nlm.nih.gov/genome/viruses/. Based on the BLAST results, we extracted only virus-associated contigs in FASTA format using the blastdbcmd program (https://www.ncbi.nlm.nih.gov/books/NBK279689/). The obtained virus-associated contigs were again subjected to BLASTN against NCBI’s nucleotide (NT) database using default parameters to delete lily endogenous virus-like sequences. Only pure virus-associated contigs were used for further analyses, such as virus genome assembly.

### Virus genome assembly

In order to assemble complete or nearly complete genomes for identified viruses, we aligned virus-associated contigs in each library on the reference virus genome using the ClustalW program implemented in the MEGA7 program [49]. The missing gaps of the virus genome were filled by mapping raw sequence reads on the virus genome using a Burrows–Wheeler Aligner (BWA) program with default parameters [50]. ORFs and 5′ and 3′ untranslated regions (UTRs) were also manually checked by comparing the corresponding reference virus genome. The assembled virus genome sequences were deposited in NCBI’s GenBank database with respective accession numbers (Table 3).

### Construction of phylogenetic trees for six identified viruses

For the construction of phylogenetic trees, virus genome sequences covering all ORFs were used. In the case of the four viruses composed of a single RNA, three LSV genomes, seven LMoV genomes, five PlAMV genomes, and three TVX genomes in this study were used. In the case of TRV composed of two RNA fragments and CMV composed of three RNA fragments, two and three different phylogenetic trees, respectively, were used. Assembled genome sequences in this study and the complete genomes of each virus available in GenBank were aligned together using the ClustalW program. The aligned nucleotide sequences were subjected to phylogenetic tree construction using the MEGA7 program with the maximum likelihood method based on the Tamura–Nei model and 1000 bootstrap replicates [49].

### Identification of SNPs for identified viruses using transcriptome data

SNPs for six viruses in each transcriptome were analyzed, as described previously [26]. In our study, we used the assembled virus genome sequence in each library as a reference genome sequence to increase SNP specificity. We aligned the raw sequence reads in each library on the identified individual viral genome using the BWA program with default parameters. We converted the Sequence Alignment Map (SAM) files into Binary Alignment Map (BAM) files using SAMtools [51]. The sorted BAM files were used to generate the Variant Call Format (VCF) file format using the mpileup function of SAMtools for SNP calling. Finally, BCFtools implemented in SAMtools was used to call SNPs. The positions of identified SNPs on each viral genome were displayed by the Tablet program [52].

## Additional file

Additional file 1: **Table S1.** Information of RNA-Seq libraries for lily cultivar “Sorbonne.” Index indicates name of individual library from three different projects, which can be further divided into A, B, and C. Raw data can be downloaded from SRA database using respective accession numbers. **Table S2.** Summary of virus-associated reads, contigs, and FPKM values. Raw sequence reads in SRA format were converted to FASTA format using the SRA Toolkit. MEGABLAST was conducted using raw sequence reads and assembled contigs against virus reference genomes using e-value 1e-6 as a cutoff. To calculate FPKM values, raw sequence reads were mapped on the reference virus genome using the BWA program followed by the pileup program implemented in the BBMap package. **Table S3.** Assembled virus genome sequences. Orange shells indicate assembled virus genomes covering ORFs, while light yellow shells indicate partial virus genome sequences. (XLSX 116 kb)

## Abbreviations

BWA: Burrows–-Wheeler Aligner
CMV: Cucumber mosaic virus
DI: Defective interfering
HTS: High-throughput sequencing
LMoV: Lily mottle virus
LSV: Lily symptomless virus
NGS: Next-generation sequencing
ORF: Open reading frame
PCR: Polymerase chain reaction
PlAMV: *Plantago asiatica* mosaic virus
PMTV: Potato mop-top virus
RNA-Seq: RNA-Sequencing
RT: Reverse-transcription
SNP: Single nucleotide polymorphism
SNP: Single nucleotide polymorphism
TBRV: Tomato black ring virus
TGB: Triple gene block
TRV: Tobacco rattle virus
TVX: Tulip virus X
UTR: Untranslated region
VCF: Variant Call Format
